# Prognostic and therapeutic roles of *SETD2* in cutaneous melanoma

**DOI:** 10.18632/aging.205894

**Published:** 2024-06-05

**Authors:** Jiani Xiong, Liping Zhu, Yunrong Fu, Zhoujie Ye, Cuimin Deng, Xinrui Wang, Yu Chen

**Affiliations:** 1 Department of Medical Oncology, Clinical Oncology School of Fujian Medical University, Fujian Cancer Hospital, Fuzhou, Fujian, China; 2 Cancer Bio-immunotherapy Center, Clinical Oncology School of Fujian Medical University, Fujian Cancer Hospital, Fuzhou, Fujian, China; 3 Medical Research Center, Fujian Maternity and Child Health Hospital, College of Clinical Medicine for Obstetrics and Gynecology and Pediatrics, Fujian Medical University, Fuzhou, Fujian, China; 4 NHC Key Laboratory of Technical Evaluation of Fertility Regulation for Non-human Primate, Fujian Maternity and Child Health Hospital, Fuzhou, Fujian, China; 5 Department of Pharmacology, College of Pharmacy, Fujian Medical University, Fuzhou, Fujian, China; 6 Department of Pharmacology, QuanZhou Women’s and Children’s Hospital, Quanzhou, Fujian, China; 7 College of Chemistry, Fuzhou University, Fuzhou, China

**Keywords:** SETD2, prognosis, therapeutic response, genomic stability, cutaneous melanoma

## Abstract

Background: Cutaneous melanoma (CM) is an aggressive form of skin cancer with limited treatment options for advanced stages. Prognostic markers that accurately predict patients’ outcomes and guide therapeutic strategies are crucial for improving melanoma management. SETD2 (SET Domain-Containing Protein 2), a histone methyltransferase involved in chromatin remodeling and gene regulation, has recently emerged as a tumor suppressor. Its dysfunction is involved in oncogenesis in some cancers, but little is known about its functions in progression and therapeutic response of melanoma.

Methods: RNA-seq and clinical data from public database were used to evaluate the survival analysis, gene set enrichment, IC50 of therapeutics and immunotherapy response. SETD2 knock-out A375 cell line (A375SETD2ko) was developed by Crispr/cas9 and CCK-8 analysis and nude mice used to evaluate the proliferation and invasion of melanoma cells *in vitro* and *in vivo*, while Western blotting tested the MMR-related protein.

Results: SETD2 was commonly down-regulated in melanoma samples which demonstrated an unfavorable survival. Cells without SETD2 expression tend to have a more progressive and invasive behavior, with resistance to chemotherapy. However, they are more sensitive to tyrosine kinase inhibitors (TKIs). They also exhibit inflamed features with lower TIDE (Tumor Immune Dysfunction and Exclusion) score and higher tumor mutation burden (TMB), showing that these patients may benefit from immunotherapy.

Conclusions: This study revealed that SETD2 dysfunction in melanoma implied a poor prognosis and chemotherapy resistance, but highly sensitive to TKIs and immunotherapy, highlighting the prognostic and therapeutic value of SETD2 in cutaneous melanoma.

## INTRODUCTION

Cutaneous melanoma (CM) is the most aggressive form of skin cancer characterized by its aggressive nature and high propensity for metastasis [1]. Despite the advancements of current treatment options like immunotherapies [2–6] and targeted therapies [7, 8], approximately half of patients fail to achieve long-lasting benefit [9–11]. The development of biomarkers that can predict treatment response, guide therapeutic decision-making, and monitor disease progression is crucial for improving patient outcomes. In recent years, significant progress has been made in identifying and characterizing biomarkers associated with cutaneous melanoma.

SETD2 was initially identified in the pathogenesis of Huntington Disease [12]. It encodes trimethylase of histone H3 lysine36 trimethylation (H3K36me3) and functions in maintaining genomic stability through mismatch repair (MMR) [13–17]. Its dysfunction has been identified in a variety of cancer types [18–22]. Studies have shown that SETD2 is necessary for homologous recombination repair and its depletion may involve in genomic instable and increasing spontaneous mutation frequency [17, 23]. SETD2 deficiency is associated with cancer occurrence and progression [22, 24, 25]. However, little is known about its function and predicting value in the development and treatment of melanoma.

In this study, we explored the data from public database and found a significant decreased level of SETD2 expression and a close correlation of it with unfavorable outcome in CM. By wound healing assay, we found that the SETD2 knocked out A375 cells exhibited a more aggressive behavior with cells arrested in G0 phase. Pathways linked to melanogenesis were enriched in SETD2 low expression CM samples and SETD2 ko A375 tumor tissue harbored by nude mice. Then treatment response in terms of chemotherapy and TKI target therapy was further evaluated using public data, while the impact of SETD2 downregulation on chemotherapy response was further investigated by IC50 scores of Cisplatin on melanoma A375 cell lines cultured with SETD2 inhibitor or knocked out SETD2. Results demonstrated that downregulation or knocking out of SETD2 was significantly associated with chemotherapy resistance but more sensitive to target therapy. Lastly, our data revealed that SETD2 mutation is closely correlated with genomic instability and ICB response. This study provides novel insights into the functional role of SETD2 in melanoma, highlighting a potential mechanism whereby SETD2 influences the prognosis of melanoma patients as well as therapeutic response.

## MATERIALS AND METHODS

### Data collection

RNA-seq data and clinical data of The Cancer Genome Atlas (TCGA), TARGET and Genotype-Tissue Expression (GTEx) were downloaded from the UCSC Xena database (https://xenabrowser.net/datapages/). The chemotherapeutic response prediction was basing on the Genomics of Drug Sensitivity in Cancer (GDSC), https://www.cancerrxgene.org/ by R package “pRRophetic”. The half-maximal inhibitory concentration (IC50) of samples was estimated by ridge regression. The batch effect of combat and tissue type of all tissues was used, and the duplicate gene expression was summarized as mean value.

### Expression analysis

Gene expression values of the transcripts from nude mice beard tumor tissue samples and TCGA database were computed by StringTie (version 1.3.3b). DESeq2 (version 1.12.4) was used to determine differentially expressed genes (DEGs) between two samples. Genes were considered as significantly differentially expressed if q-value≤0.001 and |FoldChange| ≥1.5. Gene expression differences were visualized by volcano plot. DEGs are mapped to the GO terms (biological functions) and The Kyoto Encyclopedia of Genes and Genomes (KEGG) pathway in the database, the number of genes in every term is calculated, and a hypergeometric test is performed to identify significantly enriched gene list out of the background of the reference gene list with false discovery rate (q-value) < 0.05 which was considered as significantly altered. Above analysis was performed via web-based tool platform, Sangerbox 3.0 (http://vip.sangerbox.com/).

### Survival analysis and immunotherapy response prognostic model construction

Kaplan-Meier analysis was performed to evaluate the overall survival (OS) of patients from TCGA and GTEx cohorts. For evaluating prognostic value, R software package “rms” and “regplot” using multi-factor regression model using Cox regression was used to establish a nomogram by evaluating the survival significance. P-value was conducted to assess the significance of SETD2 alteration in predicting inmmunotherapeutic response using MSKCC cohorts treated with PD-1/PD-L1 antibodies, CTLA-4 antibodies, or combo therapy. Above analysis was performed via web-based tool platform, Sangerbox 3.0 (http://vip.sangerbox.com/) [26]. The TIDE online web (http://tide.dfci.harvard.edu) was used to calculate the TIDE score.

### Cell culture and treatment

The human melanoma cell line A375 and murine melanoma cell line B16F10 were originally obtained from American Type Culture Collection (ATCC, USA). Cells were maintained in DMEM medium containing penicillin (50 U/ml), streptomycin (50 U/ml) and 10% fetal bovine serum (FBS; Gibco, USA) at 37° C in a humidified incubator containing 5% CO2. Cells were treated with 0.625, 1.25, 2.5 and 5 μg/ml of SETD2 inhibitor (EZM0414, MCE, USA) with or without cis-platinum (DDP, MCE) for 72 hours which were determined by Cell-Counting-Kit-8 (CCK-8, Dojindo, Japan) to evaluate the cell viability. SETD2 inhibitor was dissolved in dimethyl sulfoxide (DMSO). The final concentration of DMSO in each well was less than 0.1% (v/v).

### The construction of A375^SETD2ko^ cells

CRISPR-Cas9 mediated ablation of SETD2 gene was achieved with CRISPR-Cas9 RNP (provided by Haixing Bioscience, China) containing expression cassettes for hSpCas9 and chimeric guide RNA. To target exon 5~exon 9 of the SETD2 gene, two guide RNA sequence of CAGATATCAAGGCTGTATTGTGG and CCTGAGTTACTCCTGGGATGGGG were selected through the http://crispr.mit.edu website. Plasmid containing the guide RNA sequence was electrotransfected into cells using Neon transfection system according to the manufacturer’s instructions (Thermo Fisher Scientific, USA).

### Wound healing assay

A375^WT^ and A375^SETD2ko^ cells were seeded in 12-well plate (5 × 10^5^ cells/well), respectively. After cell adherence, a straight wound was made with the tip of a 200 ul pipette and cell debris was washed away with PBS. The images were acquired 0 h and 48 h of the scratch using an inverted fluorescence microscopy (Nikon, ECLIPSE Ts2R-FL, Japan). The percentage of wound healing was calculated by comparing time point 48 h with time point 0. The images were analyzed using ImageJ software. (Wound healing rate= (S_0h_-S_48h_)/S_0h_%).

### Cell cycle (propidium iodide assay)

A375^WT^ cells (1x10^6^) were collected and fixed with 75% ethanol for 4° C overnight. According to the instructions of the cycle test kit (Elabscience, China), the fixative was removed by centrifugation and incubated with RNase enzyme at 37° C for 30 min, and then stained with Propidium iodide for 30 min. The analysis was made by flow cytometry from Becton-Dickinson LSRFortessa (USA).

### Western blotting

After 72 hours of treatment with SETD2 inhibitor, cells were collected and total proteins were extracted. 20 μg protein was isolated by SDS-PAGE and then transferred to polyvinylidene fluoride membrane. After 5% skim milk powder was enclosed for 2 h, the film was incubated with the target primary antibody at 4° overnight. The membrane was then cleaned three times with TBST and incubated with appropriate secondary antibody for 2 h. Immunoreactivity was observed with ECL kit and imaged using the FluorChem M imaging system (ProteinSimple, USA). The antibodies used included: MSH2 (#ab227941, Abcam), MSH6 (#ab92471, Abcam), PMS2 (#ab110638, Abcam), SETD2 (#PA5-34935, Thermo Fisher Scientific), ATM (#ab199726, Abcam), phospho-ATM (#ab119799, Abcam), H3k36me (#ab194677, Abcam), H3k9me (#ab8898, Abcam) and GAPDH (#ab313650, Abcam). All antibodies were purchased from Abcam (USA) and used at a dilution of 1:1000.

### RNA isolation and sequencing

Total RNA was extracted using the Total RNA Extractor (Trizol) kit (B511311, Sangon, China) according to the manufacturer’s protocol. RNA integrity was evaluated with a 1.0% agarose gel. Thereafter, the quality and quantity of RNA were assessed using a NanoPhotometer ® spectrophotometer (Implen, USA) and a Qubit® 2.0 Flurometer (Invitrogen, USA). A total amount of 1 μg RNA per sample was used as input material for the RNA sample preparations. Sequencing libraries were generated using VAHTSTM mRNA-seq V2 Library Prep Kit for Illumina® following manufacturer’s recommendations and index codes were added to attribute sequences to each sample. FastQC (version 0.11.2) was used for evaluating the quality of sequenced data. (https://pan.baidu.com/s/1hdzDkQClr_itrOxM1JJJ5w?pwd=tyrk).

### Mouse model

Female nude mice at 6 to 10 weeks were acquired from the Shanghai SLAC Laboratory Animal Co, Ltd. and treated in compliance with the institutional Animal Care and the Ethics Committee approved protocol (Protocol #M018M79), accession number 2022ETKLD0115. In the early treatment model, mice were inoculated subcutaneously with 1 × 10^6^ A375^SETD2ko^ or A375^WT^ on day 0. Tumor growth was serially monitored every second day. On day 25, mice were sacrificed and tumor samples were excised for the evaluation of tumor volume.

### Statistical analysis

Data were analyzed using GraphPad Prism 9 software (GraphPad Software, USA). The data were expressed as the means ± standard deviation (SD). Statistical differences between groups were determined using t-tests, with a significance level of P < 0.05.

## RESULTS

### The expression profile and prognostic significance of SETD2 across pan-cancer samples

**Figure 1:**
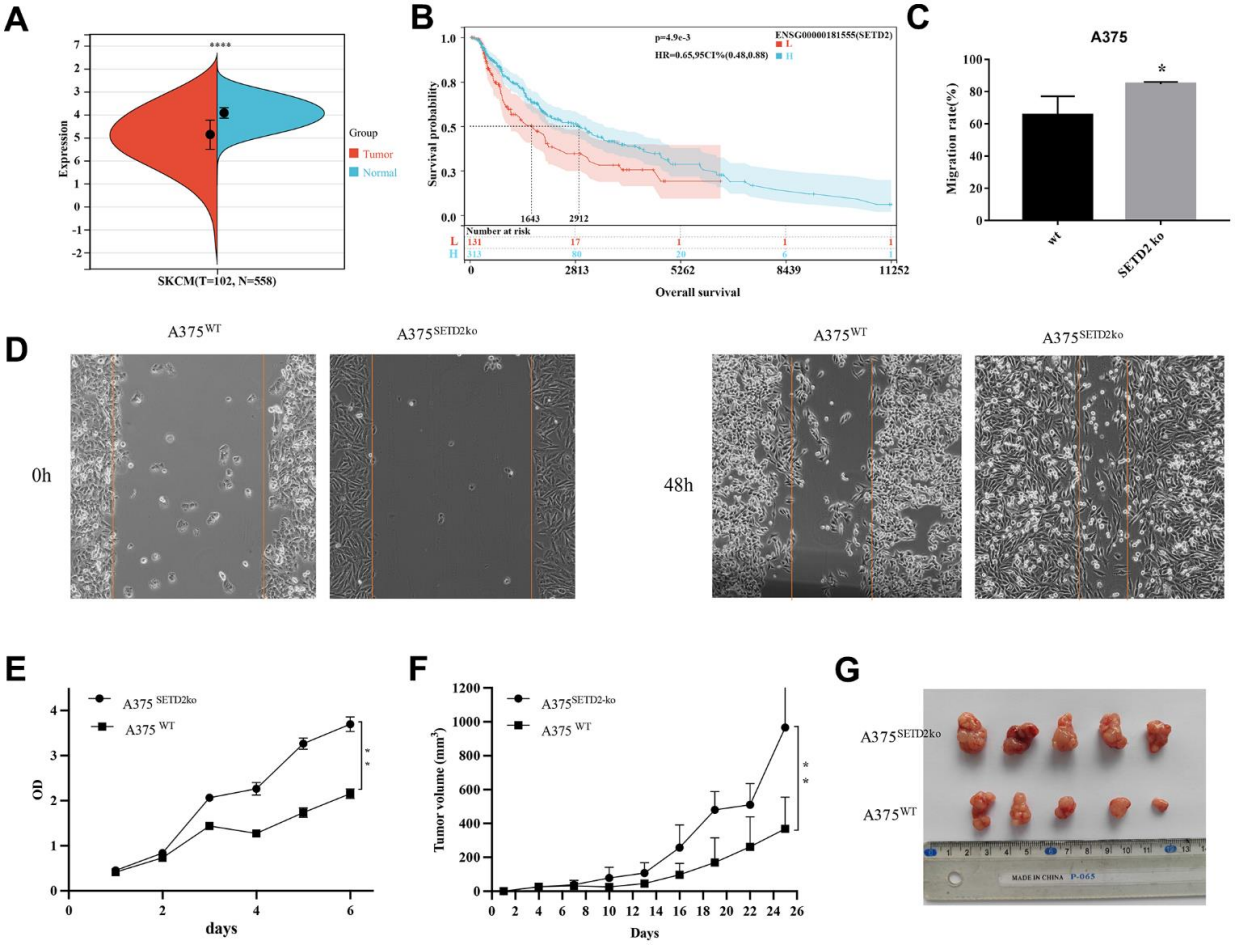
**Pan-cancer SETD2 expression and its impact on survival and migration in melanoma.** (**A**) Pan-cancer expression of SETD2 between tumor tissues and peri-tumor samples from TCGA, TARGET, and GTEx database. (**B**) Kaplan-Meier overall survival of SETD2 in melanoma from TCGA database., the best cut off value of SETD2 was taken. (**C**, **D**) Wound-healing assay compared the cell migration of A375^SETD2ko^ and A375^WT^ cells for 48h. Wound healing rate = (S_0h_-S_48h_)/S_0h_%. (**E**) CCK-8 testing the proliferation inhibition of A375^SETD2ko^ and A375^WT^. (**F**, **G**) Tumor volume of A375^SETD2ko^ and A375^WT^ cells beard by nude mice. *p < 0.05, **p < 0.01, ** *p < 0.001, the asterisk represents the degree of importance (*p). The significance of the two groups of samples passed the t-tests.

Basing on RNAseq data from skin melanoma (SKCM) samples from TCGA and GTEx (PANCAN, N=19131, G=60499) public database, we evaluated the SETD2 expression levels within cutaneous melanoma samples (Supplementary Table 1). Results found that SETD2 expression were significantly decreased in SKCM samples (Figure 1A) and samples with lower expression of SETD2 are more likely to have unfavorable overall survival outcome (Figure 1B and Supplementary Table 2). These results mean that lacking SETD2 expression may be related to tumorigenesis and progression in melanoma. Thereafter, we compared the wound healing ability of SETD2 knocked-out A375 (A375^SETD2ko^, Supplementary Figure 1) with its counterparts and found that the wound area of A375^SETD2ko^ is significantly sallower than that of A375^WT^ cells (Figure 1D and Supplementary Table 3), indicating a more aggressive bio-behavior of A375^SETD2ko^ in migration. We also evaluated the cell viability via CCK8 assay, Figure 1E showed that the OD value of A375^SETD2ko^ became higher than that of A375^WT^ cells after culturing for 2 days, demonstrating a higher cell density of A375^SETD2ko^. To further verify the underlying impact of losing SETD2 expression, we used A375^SETD2ko^ and A375^WT^, respectively, to construct nude mice tumor model and measured the tumor volume after cells were implanted for 26 days. From Figure 1F, 1G we can see that tumor volume grew in a time-dependent manner and A375^SETD2ko^ grew faster than A375^WT^ after implanted 6 days (Supplementary Table 4). These results illustrated that SETD2 plays a pivotal role in survival and tumor progression in melanoma, lacking SETD2 expression may lead to unfavorable survival outcome.

### SETD2 expression and pathways in cutaneous melanoma

**Figure 2:**
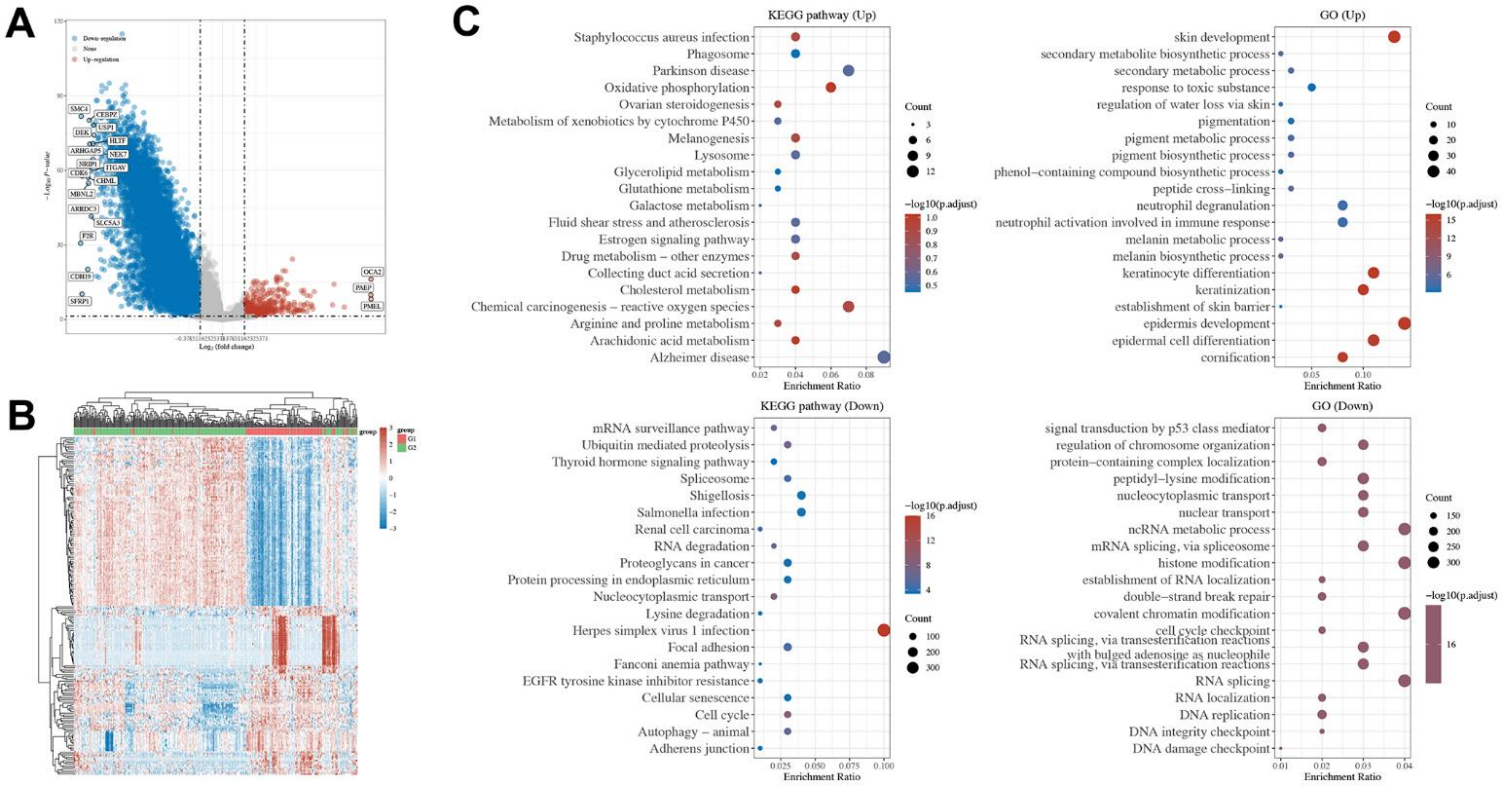
**Pathways enrichment of DGEs by SETD2 expression in TCGA-SKCM and tumor tissue of SETD2 ko A375 in mouse.** (**A**) The volcano plot exhibits the differentially expressed genes in SETD2 downregulated SKCM samples compared with its counterparts (red: upregulated genes in SETD2 downregulated SKCM samples; blue: downregulated genes in SETD2 downregulated SKCM samples). (**B**) The heatmap of differentially expressed genes in SETD2 downregulated SKCM samples compared with its counterparts. (**C**) The top enriched KEGG and GO terms by deferentially expressed genes in SETD2 downregulated SKCM samples through GSEA.

To further explore the underlying mechanisms of SETD2 downregulation in melanoma, we analyzed the significant different genes and evaluated the pathways in which SETD2 may involve using TCGA-SKCM samples. The differential genes were visualized in volcano plot, showing that compared to samples with higher SETD2 mRNA levels, 934 genes significantly up-regulated while 16 genes significantly down-regulated in samples with lower SETD2 mRNA levels (Figure 2A and Supplementary Table 5). The heatmap exhibits the top 50 differential genes with 1.5 log fold change (logFC, Figure 2B). Then we used genes with significance to enrich the SETD2 expression related pathways in KEGG and GO databases. Figure 2C shows that pathways linked to melanogenesis, skin and epidermal cells development pigment or melanin metabolic process were enriched, while pathways involving RNA metabolism and cell cycle were downregulated in SETD2 low expression group, demonstrated a possible role of lacking SETD2 expression on melanoma oncogenesis.

We also evaluated the enriched pathways in A375^SETD2ko^ tumor tissue harbored by nude mice compared with its wild-type counterparts. Supplementary Figure 2A–2C showed that genes function in RNA processing, glycosphingolipid biosynthesis series pathway and ECM-receptor interaction were mainly enriched in A375^SETD2ko^ tumor samples. Further analysis revealed that G2M checkpoint pathways, TGFb pathways and cellular response to hypoxia were positively related to SETD2 expression, emphasized the SETD2 function in oxidative-related bio-behaviors and cell cycles.

### SETD2 downregulation and DDP resistance

**Figure 3:**
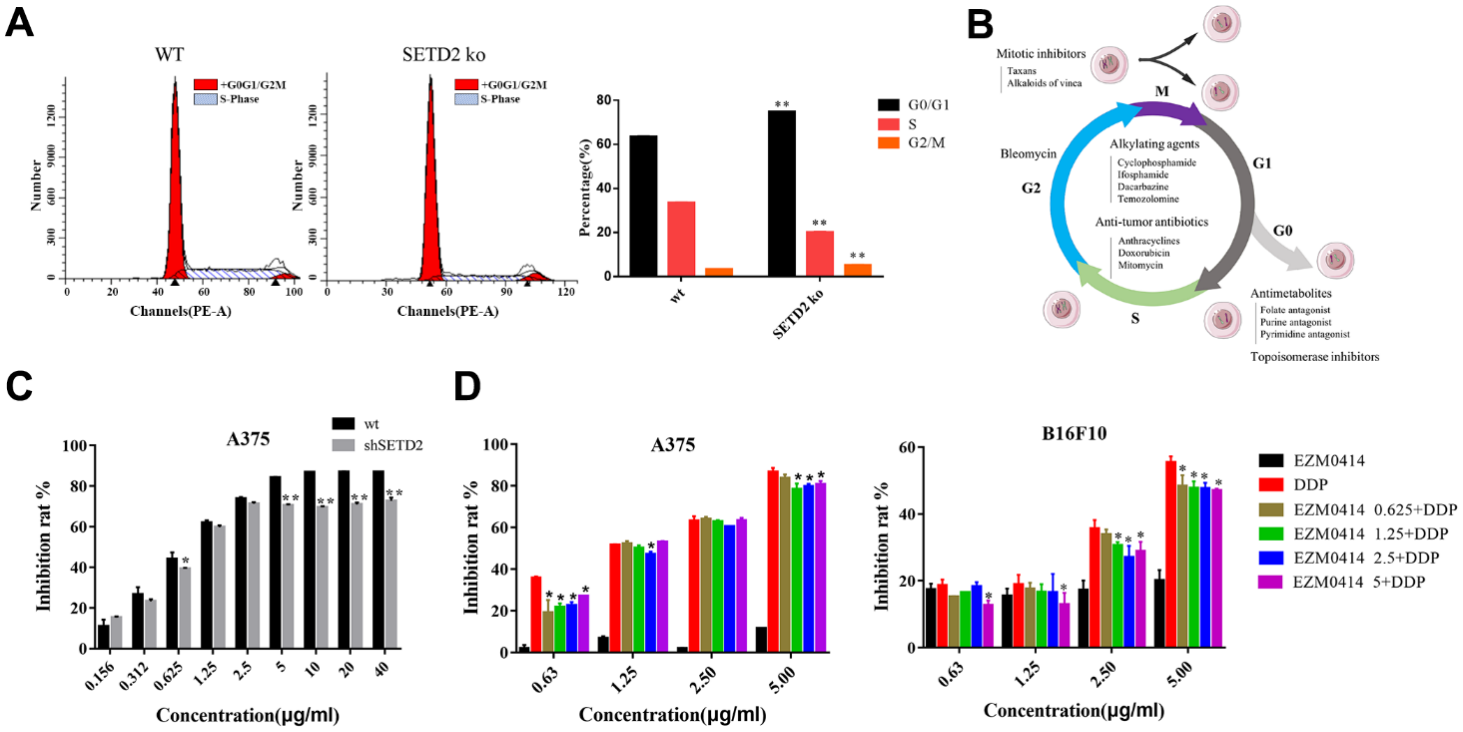
**The impact of SETD2 dysfunction on the proliferation inhibition of DDP in melanoma cell lines *in vitro*.** (**A**) The cell cycle of SETD2 ko A375 cells compared with its wild-type counterparts by flow cytometry. (**B**) The classification of chemotherapeutics. (**C**) The proliferation inhibition of SETD2 ko A375 cells cultured in DDP at 0.156, 0.312, 0.625, 1.25, 2.5, 5, 10, 20, 40 μg/ml. (**D**) CCK-8 testing the proliferation inhibition of A375 cells (left) and B16F10 cells (right) cultured in EZM0414 at 0.625, 1.25, 2.5, 5 μg/ml, DDP at 0.625, 1.25, 2.5, 5 μg/ml, then single or combination drugs were used for 72 hours.

Given that studies have shown that melanogenesis were closely related to chemo therapeutic resistance [18–22], and our data found an underlying role of SETD2 down regulation and melanogenesis. Therefore, we further evaluated cell cycles and chemotherapy response in Cis-platinum (DDP) treated A375^SETD2ko^ cells compared with widetype counterparts. The cell cycles analysis results showed that, compared to A375 cells, A375^SETD2ko^ cells tend to arrested in G0/G1 phase (Figure 3A and Supplementary Table 13). This means that A375^SETD2ko^ cells might be more tolerant to cell cycle specific agents (CCSA). As Supplementary Figure 3 showed, by exploring RNAseq data from Genomics of Drug Sensitivity in Cancer (GDSC), we found that half-maximal inhibitory concentration (IC50) of CCSA agents, such as Methotrexate, 5-Fluorouracil (Antimetabolites), Etoposide (Topoisomerase Inhibitors), Bleomycin, Paclitaxel (Plant alkaloids), Cyclophosphamide and Cisplatin for (Alkylating Agents) were higher in samples in which SETD2 expressions are significantly lower, demonstrated that they are more tolerated to these agents.

To further evaluate the efficacy of cell cycle nonspecific agents (CCNSA) on SETD2 downregulated cells, we tested the IC50 of Cisplatin (DDP) on A375^SETD2ko^ cells compared with its wild-type counterparts. Our findings showed that the proliferation inhibition of DDP on Figure 3C and Supplementary Table 6 shows that the inhibition rate of DDP were remarkably decreased in A375^SETD2ko^ cells. EZM0414, a SETD2 inhibitor that is under clinical study, were also used to analyze the impact of downregulation of SETD2 on DDP cytotoxicity. Figure 3D (left) shows that DDP alone can prevent A375 from growing in a dose-dependent manner. However, the inhibition rate decreased when A375 cells were co-cultured with DDP and EZM0414. Similar results were found in B16F10, a murine derived melanoma cell line in Figure 3D (right) and Supplementary Table 7. These results illustrate a potential role of lacking SETD2 in chemotherapy resistance in melanoma.

### SETD2 mutation and immunotherapy response

**Figure 4:**
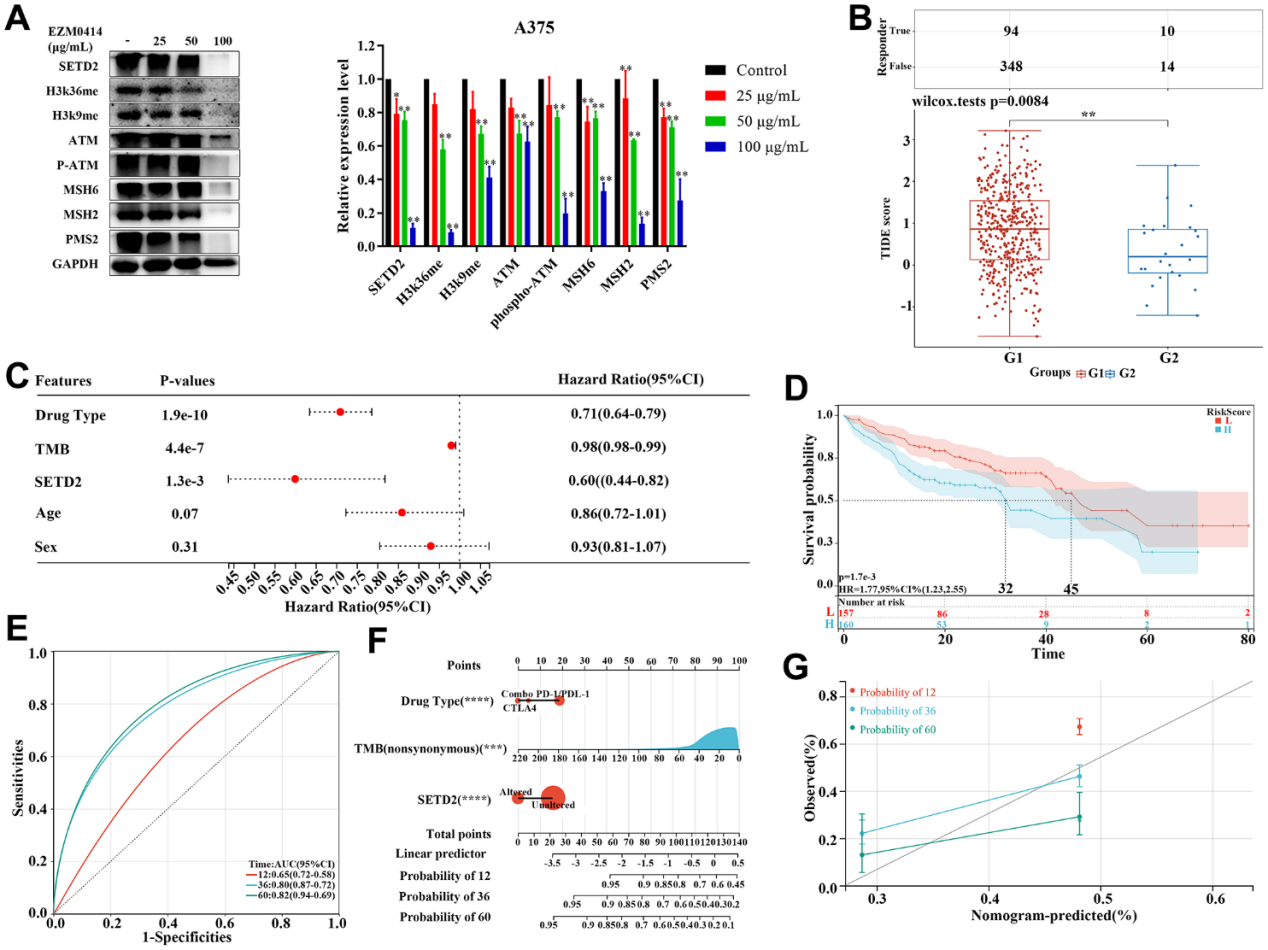
**The impact of SETD2 dysfunction on immunotherapy response in melanoma.** (**A**) Western blotting evaluated the protein level of SETD2, H3K36me3, K3K9me3, ATM, pATM, MSH2, MSH6, PMS2, and GADPH, respectively, in A375 cells cultured in EZM0414 at different concentrations. (**B**) TIDE score of TCGA-SKCM samples with different SETD2 mutation status (G1: TCGA-SKCM samples without SETD2 mutation; G2: TCGA-SKCM samples with SETD2 mutation). (**C**) The hazard ratio of clinical parameters including drug type, TMB, SETD2 mutation status, age, and sex for melanoma samples with immunotherapy from MSKCC database using Cox multivariate survival analysis. (**D**) The KM survival analysis of Cox hazards model using drug type, TMB, and SETD2 mutation status. (**E**) The ROC of Cox hazards model for predicting 1-year, 3-year, and 5-year survival. (**F**) Nomogram depicting the prognosis-predictive value of clinicopathological parameters and SETD2 status. (**G**) Calibration curves depicting the specificity and accuracy of the nomogram. *p < 0.05, **p < 0.01, ***p < 0.001.

Further evaluation by Western blotting, we confirmed that the degree of inhibition of SETD2 on A375 cells increased with the concentration of EZM0414 increased, accompanied by inhibition of histone methylation (Figure 4A). Western blotting also illustrated that EZM0414 can not only reduce the ATM phosphorylation, but the expression of mismatch repair (MMR) protein in terms of MSH2, MSH6, MIH1, and PMS2, which demonstrated the potential correlation of SETD2 and immune checkpoint blockade (ICB) response (Supplementary Table 12). Therefore, we calculated the Tumor Immune Dysfunction and Exclusion (TIDE) score of TCGA-SKCM samples to explore the impact of SETD2 downregulation on immunotherapy response. Figure 4B and Supplementary Table 8 showed that the mean value of TIDE score of SETD2 wild-type SKCM samples is higher than that of SETD2 mutant ones, illustrating a relative T cell exhaustion and exclusion in SETD2 wild-type SKCM samples but more inflamed features in SETD2 dysfunction samples. TMB levels, SNV neoantigen levels and Indel neoantigen levels were evaluated as well, showing that TMB levels, SNV neoantigen levels and Indel neoantigen levels were higher in SETD2 dysfunction SKCM samples than that in SETD2 wild-type SKCM samples. (Supplementary Figure 4A–4C and Supplementary Tables 10, 11).

In order to further verify the biomarker value of SETD2 mutation in immunotherapy in cutaneous melanoma, we analyzed the data from Memorial Sloan Kettering Cancer Center (MSKCC), in which samples had undergone immunotherapy. Multi-factor analysis showed that drug type, TMB, and SETD2 mutation are protectors for immunotherapy-treated patients with significance (Figure 4C). KM survival analysis showed a remarkable reduced overall survival in patients with higher risk score and then area under the ROC curve (AUC) is of 0.8 and 0.5 for 3-year and 5-year survival, respectively (Figure 4C, 4D). To verify the predicting value of SETD2 alteration in immunotherapy response in melanoma, we created a nomogram based on 320 melanoma patients who underwent immunotherapy from MSKCC. Standard predictors for the risk of recurrence were drug types, age, TMB levels and SETD2 alteration. This nomogram could be used to predict the individual risk of systemic relapse and to develop a risk-adapted follow-up protocol (Figure 4F and Supplementary Table 9). Remarkably, the calibration curve demonstrated a strong concordance between the predicted and observed OS rates, indicating that the nomogram effectively predicted survival (Figure 4G).

## DISCUSSION

SETD2 encodes a tumor suppressor and H3K36 methyltransferase which has been shown to play an essential role in initiating transcription and regulating DNA mismatch repair G1 and early S phase and in the maintenance of chromatin structure during elongation [27–29]. Dysfunction of this gene has been identified in a variety of cancer types and associated with an unfavorable outcome [30–33]. However, its role in melanoma initiation, progression and treatment response is understudied. Our results showed that SETD2 expression in melanoma samples was significantly correlated to melanogenesis, skin and epidermal cells development pigment or melanin metabolic process. The oncogenesis of melanoma was thought to have started from mal-pigmentation leading to melanogenesis. *In vivo* studies had revealed that the presence of melanin pigment was required by ultraviolet A-induced melanoma, which was associated with oxidative DNA damage within melanocytes and attenuated chemotherapy due to melanogenesis evolving initially [34–36]. Given that SETD2 expression also plays a role in poor prognostic outcome and chemotherapeutic resistance as shown in our study, it is highly possible that there might be a close relationship between SETD2 expression, melanogenesis, unfavorable survival and poor therapeutic efficacy. However, how SETD2 affects the prognostic and therapeutic outcomes through melanogenesis needs to be further studied.

The role of SETD2 in transcription is mainly in methylation activities. Studies have shown the loss of SETD2 influenced the inclusion of exons in genes known to be alternatively spliced and shifting in H3K36me3 signal, which leads to multidrug resistance. These data not just suggest a close relationship between trimethylation of H3K36 and RNA splicing, reflecting aberrant transcription or RNA processing [37], it also demonstrated the underlying mechanism of tumor-suppressor inactivation and multi-drug resistance via silencing SETD2 results in mRNA accumulation in the nucleus [38]. In our study, we found the significant correlation between SETD2 expression and RNA processing, we hypothesize that these pathways may be the underlying mechanisms by which the SETD2 dysfunction-related histone demethylation impacts on the survival and drug sensitivities.

Our studies have also found that SETD2 deficiency is correlated with dMMR and TMB-H and demonstrated it as a prognostic marker for immunotherapy [39]. According to the nomogram that we built up, ages, TMB, SETD2 alteration status, and immunotherapeutics are factors related to survival of patients who underwent immunotherapies. In the predicting model, TMB is a key contributor, and patients with SETD2 mutations may have higher TMB indicating the increasing likelihood of generating immunogenic tumor neoantigens recognized by the host immune system. Therefore, SETD2 mutation could be taken as a genomic biomarker to predict favorable responses to immune checkpoint inhibitors (ICIs). How SETD2 mutation results in high mutation burden has not yet been explained. Several studies have shown that SETD2 dysfunction-related H3K36me3 loss may cause its protein misfolding thus abnormal function, leading to MMR and alterations in chromatin architecture [40]. We speculate the mutation of SETD2 results in the enrichment of tumor mutation-specific neo-antigens in the cell surface, the immune system will recognize and attack these cells with the help of ICIs. The unique features of SETD2 mutation makes it a potential biomarker for cancer immunotherapy.

We observed that anti-CTLA4 monotherapy contributed less to the incidence of recurrence, followed by combo-therapy and anti-PD-1/PD-L1 mAb alone. Melanoma was the first cancer where ICBs were approved, with the anti-CTLA-4 monoclonal antibody (mAb) ipilimumab demonstrating a significant increased overall survival (OS) versus standard treatments [41, 42]. However, ipilimumab was associated with a wide spectrum and high incidence (25%) of immune-related adverse events (irAE). ICIs targeting PD1/PD-L1 axis on other hand only had 15%–20% of high grade irAE. In addition to SETD2 mutations, we found that the use of single-agent CTLA4 contributed less to the incidence of recurrence, followed by combination, and finally single-agent PD-1/PD-L1, which shows the importance of CTLA4 in the treatment of melanoma, of course, the clinical application is relatively limited compared with PD-1/PD-l1 which is mainly related to its more serious treatment-related adverse reactions [43–45]. In addition, unfortunately, the trial was not powered to compare the combination treatment versus nivolumab, therefore, which regiment to choose is still an issue that needs to be further studied.

Collectively, our findings confirmed the association of SETD2 deletion with prognostic outcome and therapeutic response in cutaneous melanoma patients, providing novel insights into the functional role of SETD2 in melanoma and a potential mechanism whereby SETD2 influences the prognosis of melanoma patients as well as treatments.

In this study, we explored the role of SETD2 in cutaneous melanoma and verified the potential value of it in predicting survival outcome and treatment responses.

## Supplementary Material

Supplementary Figures

Supplementary Table 1

Supplementary Table 2

Supplementary Table 3

Supplementary Table 4

Supplementary Table 5

Supplementary Table 6

Supplementary Table 7

Supplementary Table 8

Supplementary Table 9

Supplementary Table 10

Supplementary Table 11

Supplementary Table 12

Supplementary Table 13
