# Supplementary material for: Prognostic and therapeutic roles of SETD2 in cutaneous melanoma
Source: Aging (Albany NY). 2024 Jun 5;16(11):9692–708. doi: 10.18632/aging.205894 (PMC11210245; doi:10.18632/aging.205894)
Supplement: Supplementary Figures [file aging-16-205894-s001.pdf]

SUPPLEMENTARY FIGURES

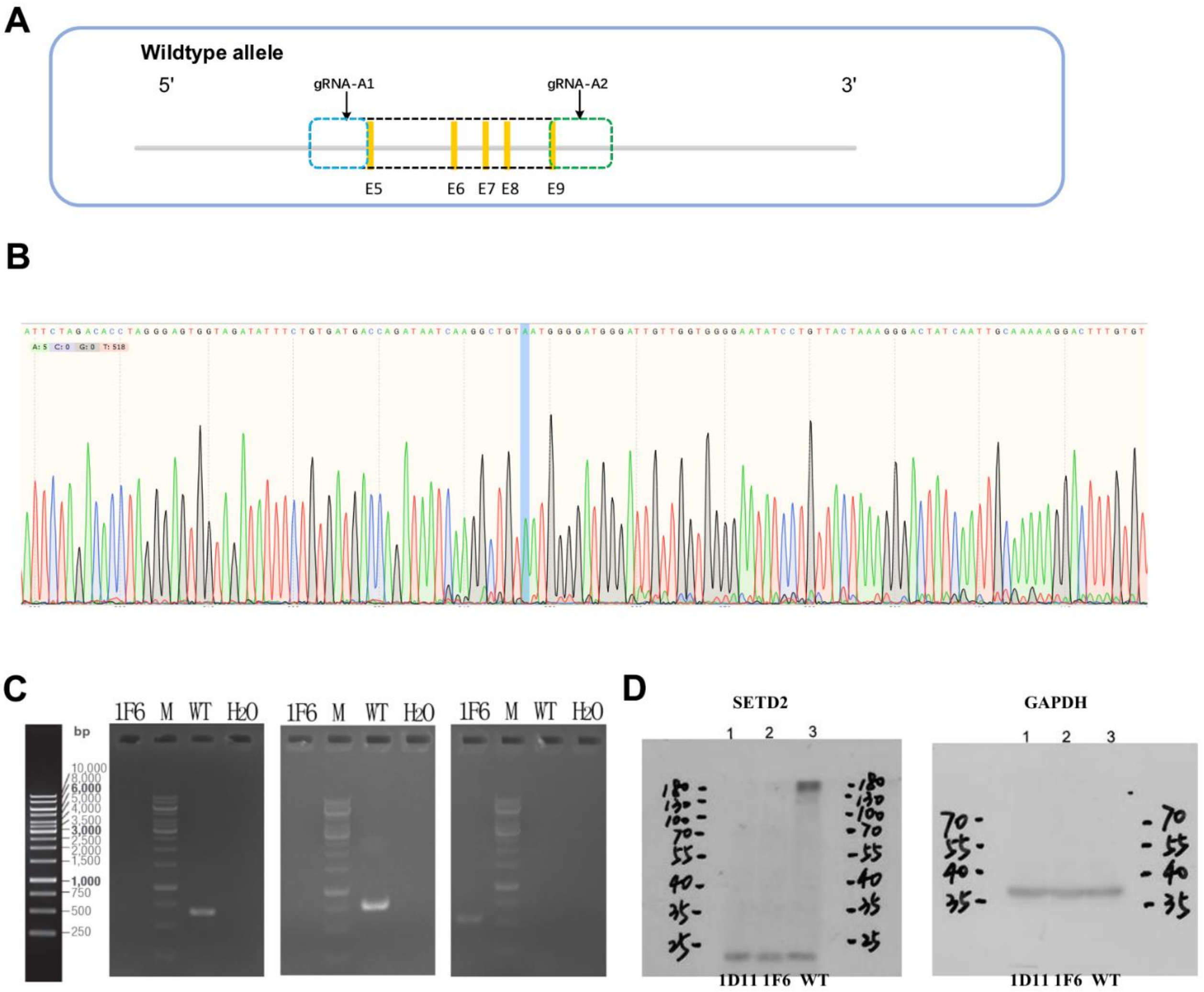

Supplementary Figure 1. The construction and the verification of A375^{SETD2ko} cell line. (**A**) Plasmid containing the guide RNA sequence. (**B**) Sequence verification of A375^{SETD2ko} cell line. (**C**) RT-PCR testing the mRNA level of A375^{SETD2ko} cell (IF6) and A375 cell (WT). (**D**) The protein level of SETD2 in A375^{SETD2ko} cells 1D11 (line 1) and 1F6 (line 2), and A375 cell (line 3). GAPDH protein levels were used as control.

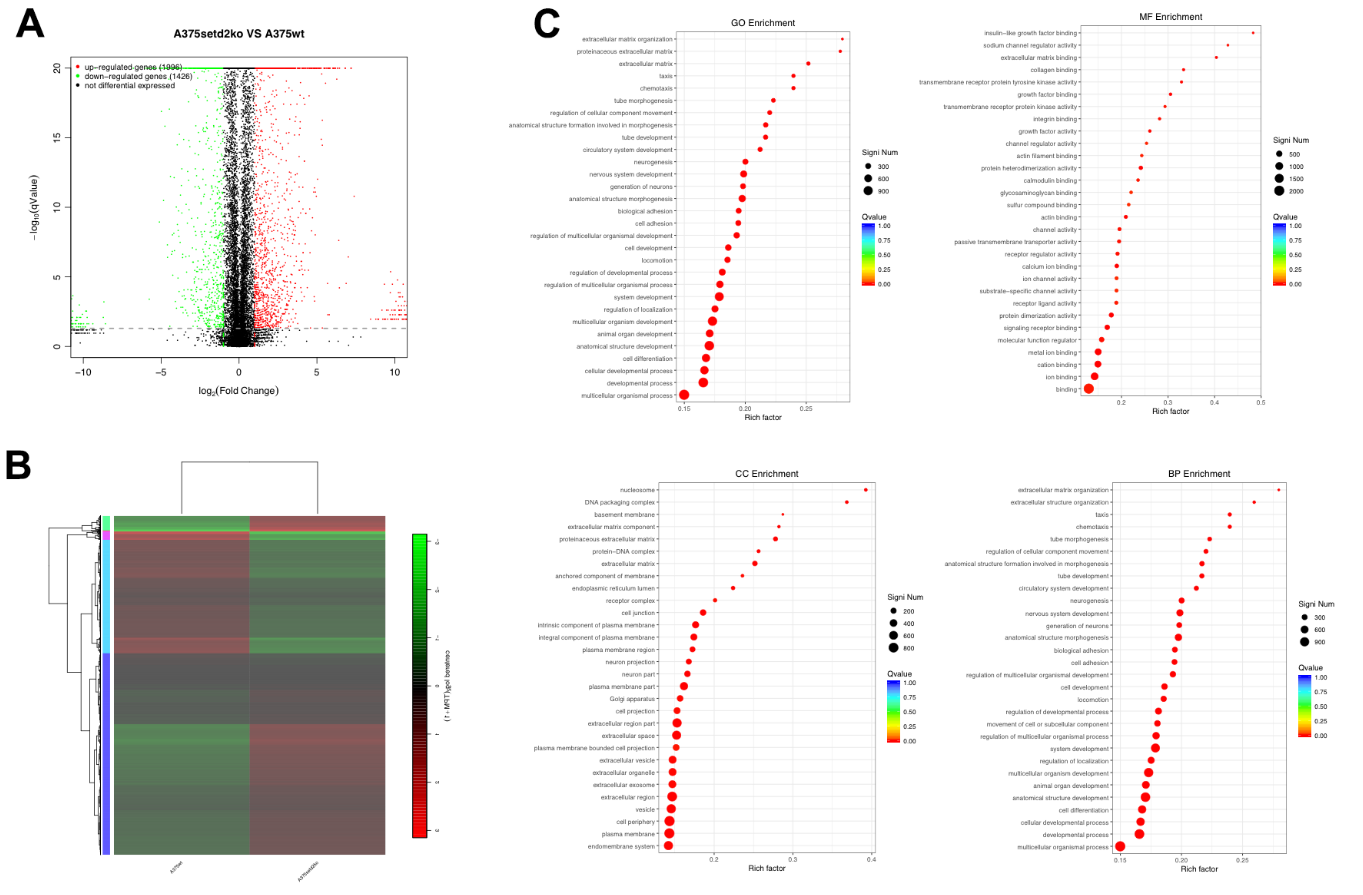

Supplementary Figure 2. Pathways enrichment of DGEs by SETD2 expression in tumor tissue of SETD2 ko A375 in mouse. (**A**) The volcano plot exhibits the differentially expressed genes in tumor tissue of SETD2ko A375 cells harbored by nude mice, comparing with A375 WT tumor tissue. (**B**) The heatmap of differentially expressed genes in SETD2ko A375 tumor tissues. (**C**) The top enriched KEGG and GO terms by differentially expressed genes in SETD2ko A375 tumor tissues through GSEA.

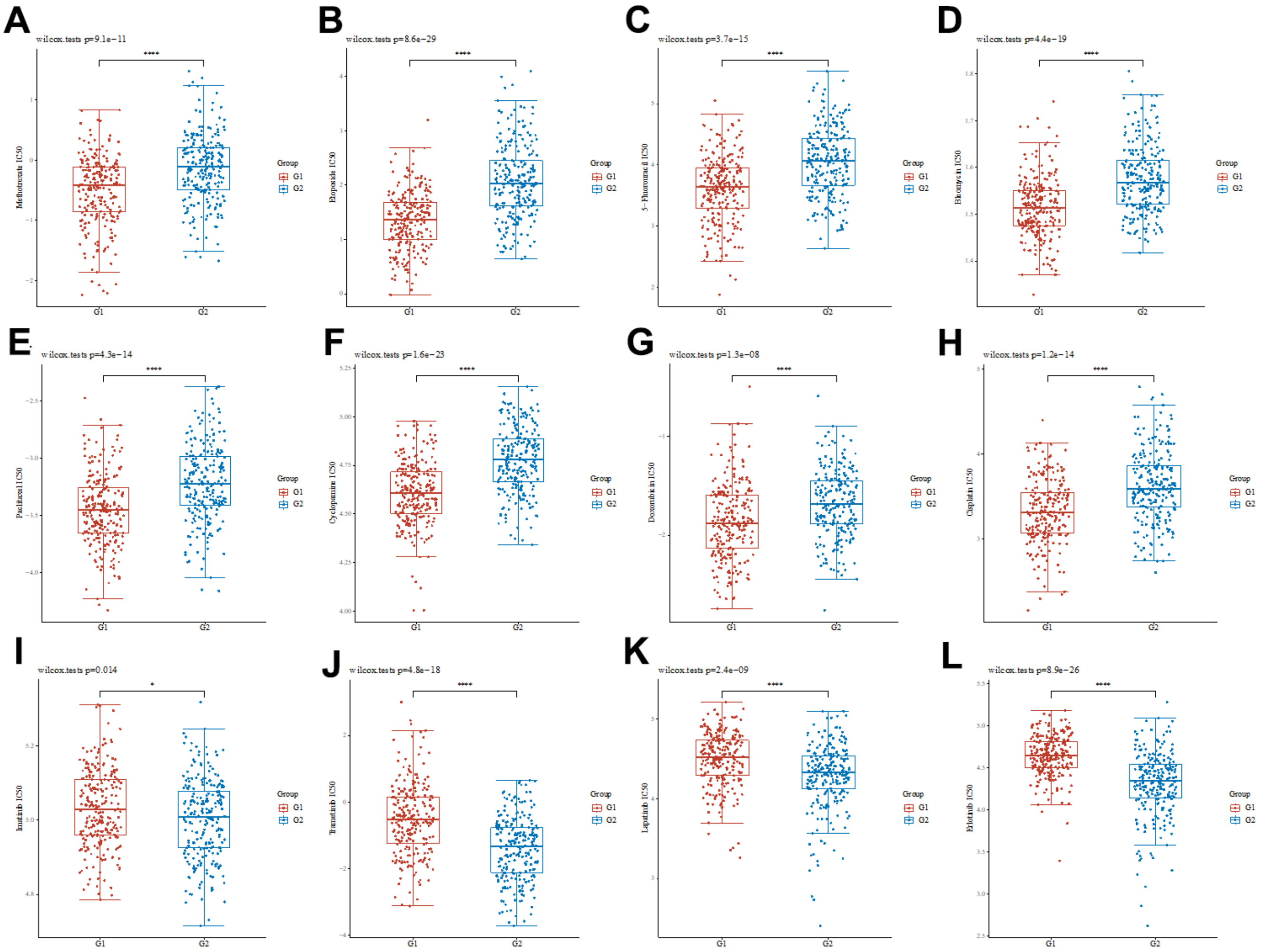

Supplementary Figure 3. The IC50 of drugs comparing SETD2 high expression group (G1, red) with SETD2 high expression group (G2, blue). (**A**–**L**) The IC50 of Methotrexate, Etoposide, 5-Fluorouracil, Bleomycin, Paclitaxel, Cyclophosphamide, Doxorubicin, Cisplatin, Imatinib, Trametinib, Lapatinib, and Erlotinib.

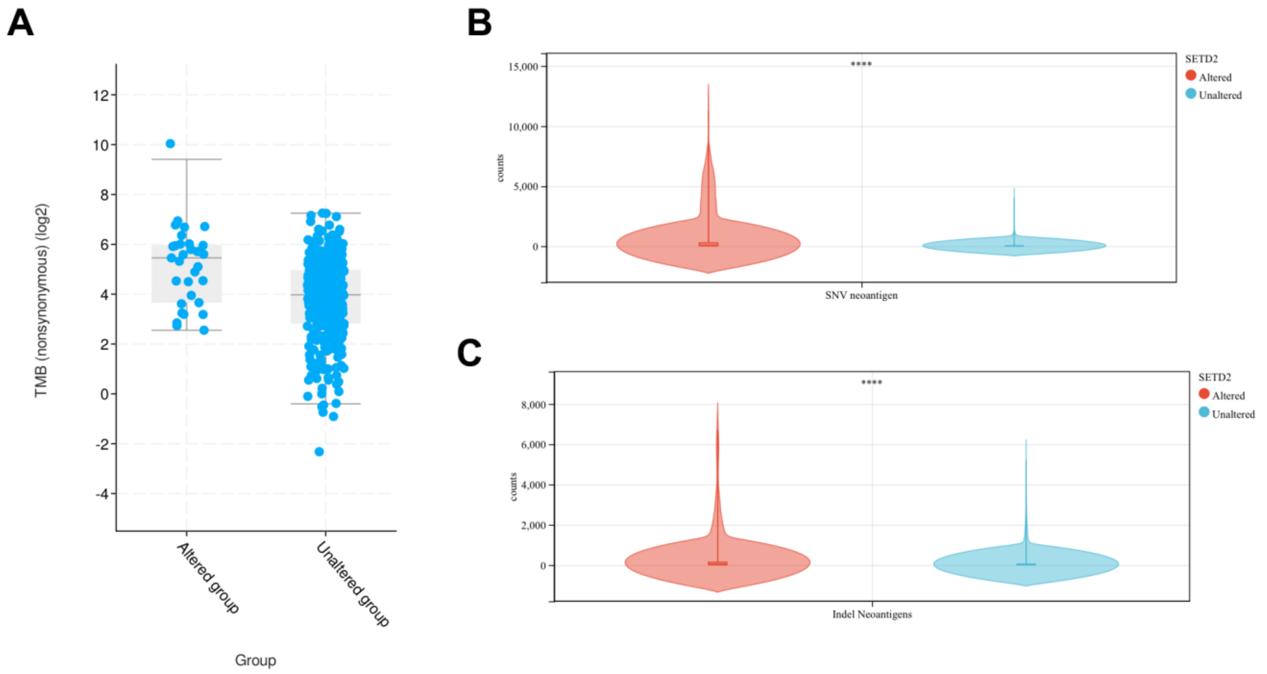

Supplementary Figure 4. The impact of SETD2 alteration on immunotherapy response in melanoma. (**A**) The TMB levels of melanoma samples comparing SETD2 altered group with unaltered ones. (**B**) The SNV neoantigen levels of melanoma samples comparing SETD2 altered group with unaltered ones. (**C**) The Indel neoantigen levels of melanoma samples comparing SETD2 altered group with unaltered ones. *$p < 0.05$, **$p < 0.01$, ***$p < 0.001$.
